# Direct visualization of hemolymph flow in the heart of a grasshopper (*Schistocerca americana*)

**DOI:** 10.1186/1472-6793-9-2

**Published:** 2009-03-09

**Authors:** Wah-Keat Lee, John J Socha

**Affiliations:** 1 X-ray Science Division, Argonne National Laboratory, 9700 S. Cass Avenue, Argonne, IL 60439, USA; 2 Current address : Department of Engineering Science and Mechanics, Virginia Polytechnic Institute and State University, Blacksburg, VA, 24061, USA

## Abstract

**Background:**

Hemolymph flow patterns in opaque insects have never been directly visualized due to the lack of an appropriate imaging technique. The required spatial and temporal resolutions, together with the lack of contrast between the hemolymph and the surrounding soft tissue, are major challenges. Previously, indirect techniques have been used to infer insect heart motion and hemolymph flow, but such methods fail to reveal fine-scale kinematics of heartbeat and details of intra-heart flow patterns.

**Results:**

With the use of microbubbles as high contrast tracer particles, we directly visualized hemolymph flow in a grasshopper (*Schistocerca americana*) using synchrotron x-ray phase-contrast imaging. *In-vivo *intra-heart flow patterns and the relationship between respiratory (tracheae and air sacs) and circulatory (heart) systems were directly observed for the first time.

**Conclusion:**

Synchrotron x-ray phase contrast imaging is the only generally applicable technique that has the necessary spatial, temporal resolutions and sensitivity to directly visualize heart dynamics and flow patterns inside opaque animals. This technique has the potential to illuminate many long-standing questions regarding small animal circulation, encompassing topics such as retrograde heart flow in some insects and the development of flow in embryonic vertebrates.

## Background

Direct visualization of blood flow in small (millimeter-centimeter scale) animals remains a major challenge. Generally, spatial and temporal resolutions better than 50 μm and 0.1 s are required for studies in insect physiology. Current techniques, including x-ray, MRI, and ultrasound or visible light probes, suffer from insufficient spatial or temporal resolutions, lack of sensitivity, or inability to penetrate the animal's opaque body. For example, MRI has recently been used to visualize the internal dynamics of circulation, respiration, and digestion in tobacco hornworm pupae, but the temporal resolution (~300 ms) and spatial resolution (~150 μm, laterally) of MRI are insufficient to distinguish fine kinematic details of motion [1]. Consequently, internal movements in small animals such as insects remain poorly understood. One prominent example is the movement of hemolymph in the insect circulatory system. Real-time synchrotron x-ray phase-contrast imaging [2] has recently been demonstrated to be an excellent technique for the study of respiratory and digestive systems in small animals, and it has the necessary resolution to study blood flow as well. This technique is capable of spatial and temporal resolutions of ~1 μm and ~100 ps, respectively, although these parameters have to be weighed against the detrimental effects on the animal because higher temporal resolutions require higher beam intensities. Despite the capabilities of synchrotron x-ray imaging, this imaging technique alone cannot distinguish blood from the rest of the soft tissue because density differences are minimal. Here, we demonstrate a new method of visualizing blood flow at the micrometer scale in intact small animals using microbubble injection combined with synchrotron x-ray imaging.

For transparent or semi-transparent animals, visible light imaging techniques have been used successfully to study gross patterns of heart activity and blood flow in small animals. Movement of hemolymph, the insect 'blood', from the abdomen to the wings of a butterfly has been visualized by introducing a visible-light dye into the hemolymph [3]. Confocal microscopy has shed new light on the heart activity and blood flow in a zebrafish embryo, showing that pumping is not peristaltic, as was previously thought [4]. Optical coherence tomography (OCT) has been used to image heartbeat in *Drosophila melanogaster *[5], and Doppler OCT has been used to visualize blood flow in frog embryos [6]. Although these recent developments are promising, they remain ultimately limited by the transmission of light: many small animals have opaque exteriors and/or the heart is located sufficiently deep that there is insufficient visible light transmission to form an image. More importantly, none of these visible light techniques have demonstrated the ability to measure intra-heart flow patterns in animals with millimeter-centimeter-scale body size.

For animals with opaque bodies, a variety of indirect, non-imaging methods have been devised to deduce heart activity and circulation patterns. To study heart activity, researchers have implanted electrodes near the heart and used the impedance between them as a proxy for heart pulsations [7,8]. This electrocardiogram technique is invasive and requires considerable skill in the implantation of the electrodes; moreover, it is not feasible for millimeter-sized animals. Alternatively, visible or infrared light transmission or reflected intensity has been used to detect heart pulsations [9,10], and by using multiple probes, it is possible to deduce the temporal relationship between different heart segments [11]. Broad patterns of hemolymph *flow *have been inferred using 'thermography' in which thermistors are attached to the exterior dorsal surface near the heart [12] and flow is deduced by small temperature fluctuations in the thermistor. Indirect techniques have also provided inferential evidence for the relationship between hemolymph flow and CO_2 _exchange [13-15], the retrograde flow through the insect heart [10,11], and for the function of accessory pulsatile organs that are hypothesized to help circulation [16]. Although these non-imaging techniques provide evidence of bulk flow, they do not provide quantitative structural information involved in heartbeat (e.g., amount of compression or shape change) or circulation (e.g., flow patterns). Furthermore, because they are inferential, these indirect techniques face common challenges involving data interpretation. For example, insects can telescope the abdomen, compress internal air sacs, and contract the gut, and these movements may occur independently from the heartbeat, making it difficult to distinguish the 'true' signal from artifact with certainty (however, see [11]). Lastly, because these are 'point' techniques, they cannot provide the contextual spatial information provided by direct imaging techniques. For example, using thermography, it is not possible to differentiate between hemolymph flow in an insect's heart versus flow in the surrounding pericardial sinus [17].

The insect circulatory system is profoundly different than its mammalian counterpart. Instead of flow occurring in a closed system of tubes and produced by a large central pump, the insect system is open, consisting of a single, open-ended tube (the heart and aorta) that runs dorsally from the abdomen to the thorax or head [18]. The tube contains slit-like incurrent and excurrent valves (ostia) that run laterally along the length of the heart. Circumferential muscles ring the heart, and fan-like alary muscles connect it laterally to the body wall. Insects pump hemolymph through the heart toward the head (anterograde flow), and, in some species, reverse the flow toward the abdomen (retrograde flow) [10,11]. However, the detailed flow patterns that result, both within the heart and body, and how exactly they are produced are almost entirely unknown. Thus the detailed mechanics of the circulatory system of perhaps the most speciose and abundant animal group on earth remains largely unknown. The ability to directly visualize the fundamental physiological functions of microfluid flow *in situ *will be extremely valuable in advancing our understanding of circulatory systems in insects and other small animals.

In this study, we visualized heart flow directly with synchrotron x-ray phase-contrast imaging combined with the use of microbubbles as high-contrast tracer particles. Microbubbles are used in clinical environments as contrast agents for ultrasound imaging [19,20]; however, the spatial resolution for *in-vivo *ultrasound imaging is wavelength limited to about 100 μm [21]. Our technique allows for direct simultaneous visualization of hemolymph flow and heart and tracheal activity with micrometer-level spatial resolution and excellent image contrast. Although MRI [1] and OCT [5] have been used to study the structural dynamics of internal heart activity in insects, these techniques do not measure actual flow rates. As far as we know, this is the first measurement of *intra-heart flow rates, patterns and dynamics *with micrometer-level spatial resolution in an insect. Although the results presented here are taken with video rate (30 Hz) acquisition, much faster exposure times and frame rates are possible at the cost of increased damage to the animal [2]. This technique extends the capability of synchrotron x-ray phase-contrast imaging to study all three essential fluid transport systems in insects: respiration (air), feeding (liquid food), and circulation (hemolymph).

## Results

**Figure 1:**
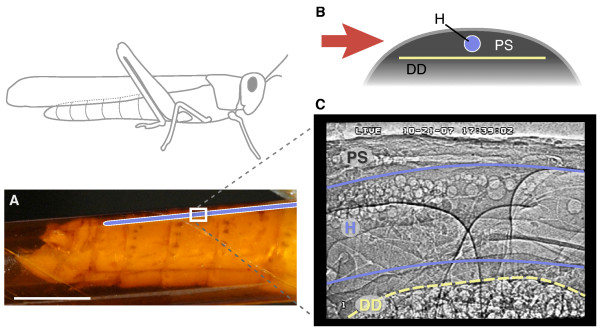
**Flow visualization in the heart of a grasshopper (*Schistocerca americana*) using synchrotron x-ray phase-contrast imaging**. (A) Side view of the grasshopper abdomen showing the approximate location of the heart (blue) and the relative size of the imaging window (white rectangle, 1.3 × 0.9 mm). The abdomen is encapsulated in an x-ray transparent Kapton tube. Scale bar, 5 mm. (B), Cross-sectional schematic of the dorsal abdomen showing the relative sizes and locations of the heart (H), dorsal diaphragm (DD), and pericardial sinus (PS). The red arrow indicates the orientation of the x-ray beam. (C) X-ray video still of a region in the dorsal 3rd abdominal segment in lateral view. Round structures are air bubbles used to visualize patterns of heartbeat and hemolymph flow.

Microbubbles ranging from 10–150 μm in diameter were clearly visualized, as were air sacs and numerous tracheae, including a main longitudinal tracheal trunk that runs adjacent to the heart (Fig. 1C and Additional files 1, 2, 3, 4, 5, 6 and 7). The buoyant bubbles accumulated along the ventral edge of the dorsal diaphragm, which partially compartmentalizes the dorsal heart region. Within the heart, the buoyant bubbles demarcated the dorsal edge of the heart lumen. The ventral edge of the heart lumen could not be clearly identified, but the paths of microbubbles during flow within the heart suggested its ventral extent. Microbubble flow, compression of the pericardial sinus and changes in the tracheal diameters were all clearly visualized (Additional files 2, 3, 4, 5, 6 and 7).

**Figure 2:**
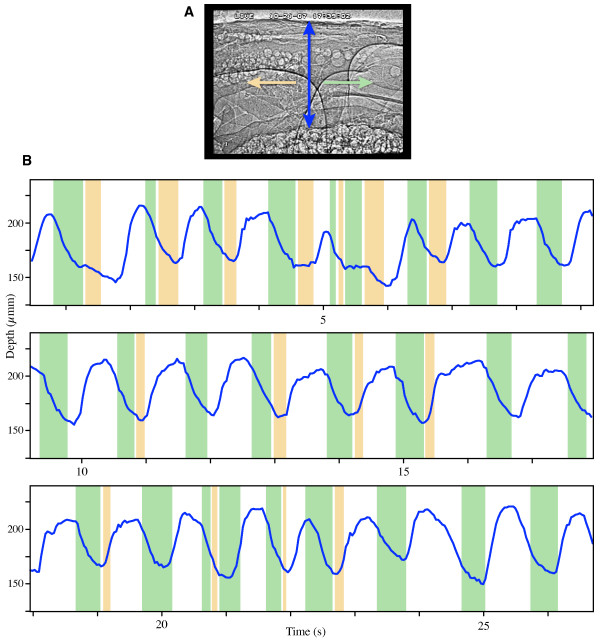
**Relationship between heart movements and hemolymph flow (3rd abdominal segment) in the grasshopper *Schistocerca americana***. (A) Side view x-ray video image indicating the color conventions used in (B). Anterior is to the right; dorsal is to the top. (B) Time series from one video sequence showing the relationship between pericardial sinus depth (blue) and hemolymph flow direction (green, yellow, white). Decrease in pericardial sinus depth corresponds to compression. Green represents net forward (anterograde) flow, yellow represents backflow, and white indicates zero flow. Average compression frequency was 55 cycles/min (0.92 Hz). In most instances, forward flow occurred only during compressive movements of the pericardial sinus, and presumably of the heart itself. Backflow did not occur with each forward flow event, but when it did, it occurred directly after the forward flow event. Note that there was no observable hemolymph movement for roughly half the time.

**Figure 3:**
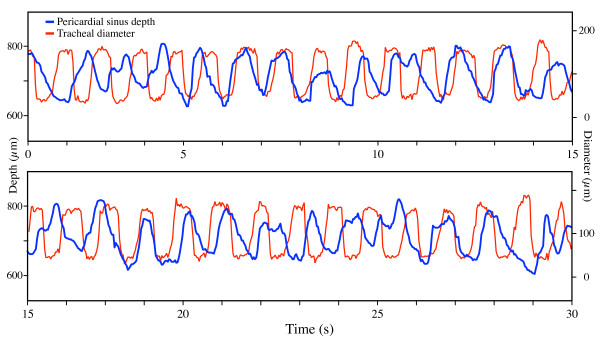
**Relative timing of compression of the pericardial sinus (blue) and a main longitudinal trachea (red) in the dorsal abdomen (4th segment) of the grasshopper *Schistocerca americana***. This sequence shows that the movements of the pericardial sinus and the trachea are not phase locked.

As seen within the field of view (1.3 H × 0.9 V mm), the flow patterns were complex and dependent on location. At any particular location along the heart, general flow patterns were repetitive, but not strictly time-periodic, over the course of the measurement (10's of seconds). For most pulsations (e.g., Fig. 2), forward flows coincided with local pericardial compressions (see Methods). Interestingly, back flows, when they occurred, began *during *pericardial compression, and ended at or after the end of the compression. However, this could be due to a phase lag between the hemolymph and the bubble (see Discussion). The amplitude of pericardial sinus compression varied locally (Additional file 2). Compressions of the longitudinal tracheal trunk were not synchronized with the pericardial sinus compressions (e.g., Fig. 3); however, their frequencies were similar (0.8–1.0 Hz). Air sac compressions also appeared to be independent of pericardial sinus compressions (Additional file 1). Flow within the heart was pulsatile, but did not appear to be bolus-like. Unlike previously observed peristaltic transport in the gut [2] in which the bolus-like transport of food and gut peristaltic waves were readily apparent, the hemolymph transport mechanism within the heart remains unknown from this preliminary study.

**Figure 4:**
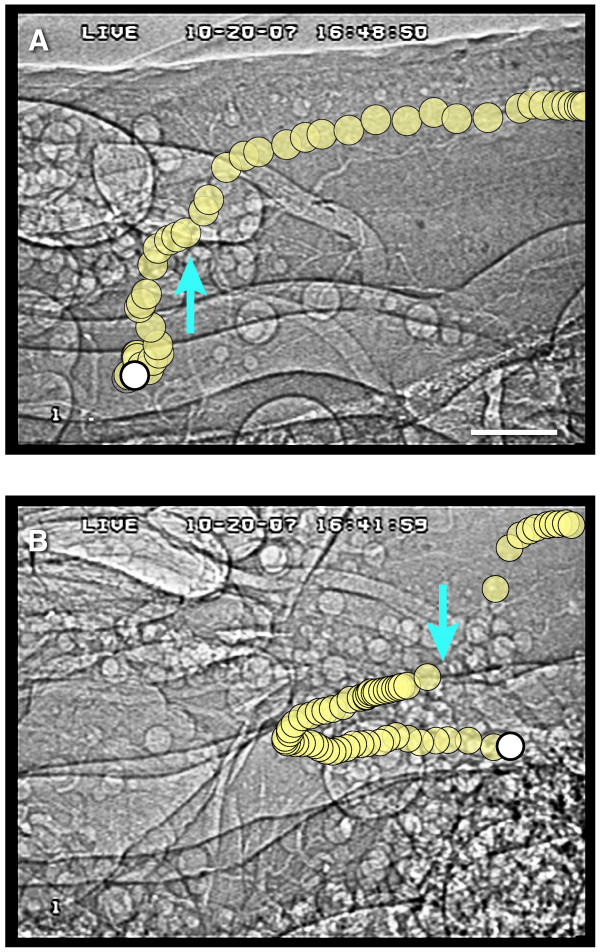
**Examples of bubble movement through presumed ostial openings in the heart wall of the grasshopper *Schistocerca americana***. Yellow bubbles represent single bubbles tracked through one video sequence (30 Hz), with the start of the sequence indicated in white and bubble positions corrected for whole-body movement. The blue arrows represent the apparent locations of ostia, inferred from the movements of the bubbles. (A) Heart entrance event in which the bubble traveled dorsally and anteriorly in the pericardial sinus, moved through the ostium, then was transported anteriorly within the heart (5th abdominal segment). For corresponding video, see Additional File 6. Scale bar, 200 μm. (B) Entrance event in which the bubble moved posteriorly in the pericardial sinus, dorsally through the ostium, and anteriorly after entering the heart (6th abdominal segment, same individual as above). For corresponding video, see Additional File 7.

**Figure 5:**
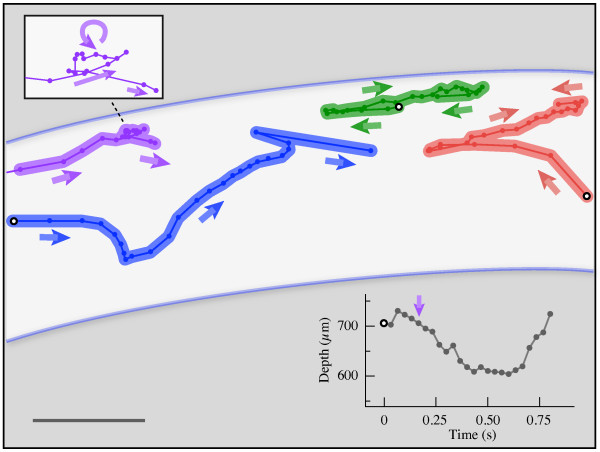
**Examples of non-uniform flow patterns in the heart of the grasshopper *Schistocerca americana***. This schematic depicts the paths of four bubbles digitized in one video sequence (duration, 0.83 s) moving within the heart (white background, 6th abdominal segment). Each color represents a different bubble. Time between points is 0.033 s; the start of the sequence is indicated with black and white circles. The overall motion of the flow is from left to right (anterograde; toward the head), but the microscale flow is non-uniform. For example, the red and green bubbles begin moving to the left while the blue bubble was moving to the right; these bubbles also reverse direction to the left at the end of their sequences. Note that the purple bubble displays a counterclockwise vortical movement (shown with greater detail, inset box). The corresponding movement of the pericardial sinus is depicted in the graph (lower right). Note that the purple bubble enters the frame from the left at t = 0.20 s, 5 frames later than the other bubbles. Scale bar, 200 μm.

The power of this technique is exemplified in the ability to track small-scale flow patterns. For example, bubbles can apparently be seen to enter the heart by moving through incurrent ostia (Fig. 4, Additional files 6 and 7). However, it was not possible to differentiate if the incurrent ostia were opening actively or passively, nor were we able to positively identify movement of bubbles out of the heart through excurrent ostia [22]. The local flow patterns near the ostia, but external to the heart, were complex. In general, bubbles were observed to enter the ostia originating both posteriorly (e.g., Fig. 4a, Additional file 6) and anteriorly (e.g., Fig. 4b, Additional file 7). In either case, once they entered the heart, the bubbles were swept anteriorly with the heartflow. Another example shows the complexity of flow within the heart. In Figure 5, four microbubbles are tracked over the course of 25 frames (~0.83 s) in one section of the heart. This sequence suggests that the bubbles were pulled towards the middle of the image during diaphragm dilation, and upon compression, hemolymph was transported towards the head. The ability to image individual microbubbles allowed us to quantify instantaneous velocities and potentially to map complex flow fields. Such detailed flow information cannot be obtained from any other technique.

Although the main purpose of this paper is to present this new technique, our measurements allow us to make a few preliminary observations on the hemolymph flow in the heart of the grasshopper *Schistocerca americana*.

1. In general, flow patterns are complex, and are time and location dependent. Furthermore, some flows appear to be three-dimensional. In some observations, there appeared to be no overall transport, but simply a back-and-forth oscillation of the hemolymph (Additional files 4 and 5). This preliminary study suggests that the origin of hemolymph transport may be more complicated than the commonly assumed peristaltic motion [10,11,14].

The maximum speed of bubbles that could be tracked was 9.5 mm/s; faster moving bubbles were observed but could not be reliably measured due to motion blur (exposure, 16 ms). Based on this maximum speed, a heart diameter of 0.5 mm, and using the viscosity of water as an estimate of that of hemolymph, we calculate a Reynolds number of 4.8, suggesting that flows are in the laminar regime.

3. For two individuals that were imaged for 20–30 minutes each, there was no evidence of a sustained retrograde fluid transport. The microbubble motion was either pulsatile with overall forward transport, or oscillatory with no net transport.

4. Respiratory structures (tracheae and/or air sacs) were compressed in patterns both synchronous and asynchronous to the local heartbeat.

5. No immediate detrimental effects on the animal were observed, either from the x-rays or from the microbubbles. The animals that were injected with microbubbles and under x-ray irradiation of the heart for up to an hour were alive 24 hours later. This is consistent with our previous results [2] which showed that there were no observable negative effects in insects at similar x-ray intensities under irradiation of the abdomen. Local x-ray damage must have occurred, but effects on heart, tracheal, or abdominal movements and behavior were not apparent within this time frame.

## Discussion

Prior work on grasshopper hearts consists of optical cardiography (*Schistocerca gregaria*, [9]), electrocardiography and mechanography (*Melanoplus differentialis*, [23]), and direct observation through live dissection (multiple species, [22]). These studies quantified heart activity, but did not measure hemolymph flow patterns within the heart. One valid comparison that can be made among studies is between mechanography (pulsations detected using an electromechanical setup) and our measured pericardial compressions. Among studies, measured pulsation frequencies (0.8–1.0 Hz) and waveform shapes are similar. Additionally, Jahn and colleagues [24] showed that the shapes and frequencies of the optical, electrical and mechano cardiography signals can vary greatly, from individual to individual, and show large dependence on temperature, further reinforcing the need for understanding the link between electrocardiography and actual flow.

More generally, we can compare this x-ray imaging method with other techniques that have recently been used to study small animal circulation. Confocal microscopy provides 2D depth-resolved images with micrometer-level spatial resolution, with depth of focus usually no more than a few micrometers, and high speed confocal microscopy has been demonstrated at 1000 frames per second (fps) [25]. Microscopic flow patterns can be quantified through the use of fluorescent tags, although depth of focus can be a limitation if the flow is not parallel to the focal plane. OCT provides cross-sectional images of the sample with spatial resolution in the tens of micrometers [26], and framing rates of 1000 fps have also been demonstrated [6]. Doppler OCT can provide information on bulk flow, but it is not quantitative [6]. Both confocal microscopy and OCT are visible light techniques and therefore are limited by light transmission. Recently, MRI has been used to image the cross-sectional structure and flow in the pupae of the tobacco hornworm caterpillar (*Manduca sexta*) [1]. However, the spatial resolutions (156 μm with a slice thickness of 500 μm) are still not comparable to synchrotron x-ray phase contrast imaging or visible light techniques. MRI cross-sectional images can be obtained at a frequency of about 3 Hz [1]. In contrast, the x-ray technique described in this paper has spatial resolution that is comparable to visible light techniques (μm-level), and high-speed x-ray imaging is possible as well. Compared to visible light techniques, x-ray imaging has the advantage of high penetration and can be used for opaque samples. X-ray images are 2D transmission projections and therefore depth information is lost, but this has the advantage that one can see through the entire animal without the need to adjust a 'depth of focus'. Transverse flow (perpendicular to x-ray direction), when imaged with tracer particles, can be quantified at the micrometer-level, which cannot be done with MRI or OCT. Two disadvantages of this technique compared to visible light or MRI are that (1) the x-rays can have a detrimental effect on the animal [2] and (2) the need for contrast agents or tracer particles.

Several challenges and improvements need to be addressed to make this technique more accessible. First, it is necessary to tailor the contrast agent to the animal or species. In particular, it is important to limit the amount of bubbles within the volume of interest; otherwise, the projected overlapping bubbles will overwhelm specific features of interest. Second, it is important to generate bubbles of the proper size; obviously, for visualization of internal heart flow, the sizes should be substantially smaller than the cross-sectional heart dimensions. Third, different species may require different injection protocols depending on anatomy and physiology. Finally, there is the question of bubble buoyancy and differences between bubble and fluid motion, which may be a major limitation on the quantification of flow. In our experiments, we oriented the insect's abdomen horizontally so that any observed posterior-to-anterior bubble flow could be definitively attributed to fluid flow, rather than to buoyancy. The buoyancy issue can be addressed in future studies by using tracer particles that have a more comparable density with hemolymph, such as metal-coated hollow glass spheres. Differences between actual fluid motion and bubble/tracer particle motion, and the effects of the bubbles themselves on the fluid motion, are complex questions that are beyond the scope of this paper, especially in the realm of non-uniform pulsating flow that we see here. Nonetheless, for the parameters used here, the speed of the bubble should reflect the speed of the fluid, although there may be a phase difference between the two [27,28]. The use of neutrally buoyant and smaller tracer particles should solve this problem. Finally, although we were able to quantify representative instances of individual bubble speed, the frame rate (30 fps) of our camera was not fast enough for a more complete velocity map. Planned future work includes the use of a high speed video camera (> 500 fps), which should enable us to directly measure peak hemolymph flow velocities; the addition of flow-through respirometry, which would allow further insight into circulation-respiration coupling; and combination of this technique with available indirect techniques (e.g., thermography, light transmission) for purposes of verification and for generating complementary data.

## Conclusion

For the first time, detailed fluid flow patterns in small animals can be imaged at the micrometer scale. In addition to insects, this technique should find applications in areas including vertebrate development (in such model systems as zebrafish or chick), and any other field in which the understanding of moving fluids behind opaque anatomy, at small scales, is key to answering outstanding questions of physiology. The potential applications of this technique are very broad.

## Methods

*Schistocerca americana *grasshoppers (mass, 1.3–1.7 g) were obtained from the laboratory colony of Prof. Jon Harrison (Arizona State University) and were supplied with food and water *ad libitum *prior to the experiment. Only males were used. We used clinical grade microbubbles (lipid/octafluoropropane bubbles; Definity, Bristol-Meyers Squibb) as the contrast agent. The average size of the microbubbles in the contrast agent (98% < 10 μm, 2.4–3.1 μm median) was near the spatial resolution of our system and therefore was at the limit of visibility. These clinical grade microbubbles could indeed be seen in the grasshopper, but they did not provide the degree of flow visualization that we were seeking. To achieve larger, more easily tracked bubbles in the animal, we purposely left air (roughly the same volume as the Definity solution) in the tip of the needle when loading the syringe with the microbubble solution; during injection, both air and solution were then transferred into the grasshopper, creating additional 10–150 μm sized bubbles in the hemolymph. For the creation of the larger bubbles, the Definity solution merely acted as a non-toxic surfactant to reduce surface tension, and it is likely that other cheaper alternatives may work as well. We experimented with different injection protocols; for the data presented in this paper, we injected the animal both on the dorsal and ventral sides of the abdomen, near the third to last segment. On the dorsal side, we attempted to inject into the pericardial sinus, but were unable to verify placement due to hemolymph circulation between the time of injection and the start of imaging. Bubbles appeared stable over the course of the measurements (20 min to 1 hr), and disappeared within 24 hours.

Animals were held in place with a clip attached to the wings, with the abdomen oriented parallel to the ground and orthogonal to the x-ray beam, providing a lateral view image (Fig. 1). The legs and abdomen were inserted into separate Kapton (Dupont) tubes with slightly larger diameters, permitting small movements but precluding large-scale movements for stable imaging. The tubes were arranged to position the legs out of view. The animal was mounted on a translation stage, allowing movement relative to the x-ray beam, and translation was controlled remotely.

For x-ray imaging, the x-ray energy was 25 keV and the incident power density was 80 W mm^-2^, similar to that used in our previous work [2]. The x-rays passing through the insect were converted to a visible light image using a cerium doped Y_3_Al_5_O_12 _scintillator, which was then imaged onto a video camera (Cohu 2700) using a Mitutoyo 5× microscope objective together with a tube lens. The field of view was approximately 1.3 mm × 0.9 mm. Video images were recorded onto miniDV tapes. iMovie (Apple, OSX) was used to convert movie clips into still image stacks (30 frames per second) and then analyzed using MATLAB (Mathworks, MA, USA). A 400-mesh transmission electron microscope grid was used for image calibration. The data were collected at the X-ray Operations and Research beamline 32-ID-C at the Advanced Photon Source, Argonne National Laboratory.

For image analysis, the following protocol was used. To quantify pericardial compression, we identified locations along the heart with the most pronounced movement and digitized two points: one on the dorsal diaphragm, which moved dorsoventrally during compression, and one near the dorsal cuticle, which was static relative to compression. We defined pericardial depth as the vertical distance between these points; decreasing pericardial depth indicates compression. For tracheal compression analyses, we measured the diameter of the trachea by digitizing the dorsal and ventral edges of the tube. For some sequences, we scored flow discretely as forward (bubbles moving anteriorly), backward (bubbles moving posteriorly) or none (no overall translation of bubbles). Due to bubble buoyancy, our flow quantification was restricted to the dorsal regions of the heart.

## Authors' contributions

Both authors contributed equally to the conception, data collection, analysis and writing of this work, and have read and approved the final manuscript.

## Supplementary Material

Additional file 1 **Synchrotron x-ray phase-contrast video of heartbeat in the grasshopper *Schistocerca americana*.** Movie depicts the grasshopper's 8th abdominal segment in lateral view, with dorsal oriented upward and anterior to the right. Field of view is 1.3 × 0.9 mm. The dorsal edge of the abdomen can be seen at the top. The diagonal lines running from upper left to lower right are margins of the x-ray transparent Kapton tube. In this sequence, pulsatile movements in the dorsoventral axis associated with heartbeat can been seen, but flow within the heart is not apparent due to lack of contrast between hemolymph and the surrounding tissue. Large collapsing circular structures are air sacs. Tracheal tubes are also visible, particularly the two main tubes running horizontally at the bottom of the frame. Click here for file

Additional file 2 **Typical hemolymph flow in the heart of the grasshopper *Schistocerca americana *I.** Movie depicts the grasshopper's 3rd abdominal segment in lateral view, with dorsal oriented upward and anterior to the right. Field of view is 1.3 × 0.9 mm. The dorsal edge of the abdomen can be seen at the top. The diagonal line in the lower left corner is a margin of the x-ray transparent Kapton tube. In this video, bubbles can be seen in three locations: within the perivisceral sinus, collected en masse and abutting the dorsal diaphragm (lower third of image); within the heart, moving rapidly left and right; and surrounding the heart in the pericardial sinus, either static or moving less rapidly than within the heart. The dorsal diaphragm moves dorsoventrally in association with heartbeat. A main tracheal trunk running antero-posteriorly is also compressed in association with these movements, but not strictly so. Within the heart, flow is complex and non-uniform, as evidenced by the trajectories of the bubbles. There is net transport of the hemolymph anteriorly, but the bubbles can be seen moving posteriorly as well. Additionally, vortical trajectories of bubbles can be seen. Due to bubble buoyancy, most bubble trajectories in the heart occur in dorsal margin. Click here for file

Additional file 3 **Typical view of hemolymph flow in the heart of the grasshopper *Schistocerca americana *II.** Movie depicts the grasshopper's abdomen in lateral view, with dorsal oriented upward and anterior to the right. Field of view is 1.3 × 0.9 mm, mostly of the 5th abdominal segment, although the start of the 4th can also be seen on the right side of the image. The dorsal edge of the abdomen can be seen near the top, and the abdominal segment boundary appears on the right. The diagonal lines running from lower left to upper right are margins of the x-ray transparent Kapton tube, whose outer boundary can be seen at the top. This video shows net transport of hemolymph in the heart toward the right (anterograde flow), but intermittent backflow to the left can be seen as well. Note that unlike in Additional file 2, the main tracheal trunks are not compressed along with the pulsatile movements. Click here for file

Additional file 4 **Oscillatory hemolymph flow in the heart of the grasshopper *Schistocerca americana *I.** Movie depicts the grasshopper's 3rd abdominal segment in lateral view, with dorsal oriented upward and anterior to the right. Field of view is 1.3 × 0.9 mm. The dorsal edge of the abdomen can be seen near the top. The diagonal lines running from upper left to lower right are margins of the x-ray transparent Kapton tube; the spotting in the upper right corner is due to residual material on the tube wall. In this video, the large bubbles in the heart enter the frame from the left, reverse direction, and exit to the left, demonstrating oscillatory flow with no net transport. Also note the slower, steadier movement of much smaller particles near the dorsal abdominal wall. These are the injected Definity microbubbles, which appear to be located within the pericardial sinus that surrounds the heart. Click here for file

Additional file 5 **Oscillatory hemolymph flow in the heart of the grasshopper *Schistocerca americana *II.** Movie depicts the grasshopper's 6th abdominal segment in lateral view, with dorsal oriented upward and anterior to the right. Field of view is 1.3 × 0.9 mm. The dorsal edge of the abdomen can be seen near the top. The diagonal lines running from upper left to lower right are margins of the x-ray transparent Kapton tube. This video demonstrates a second sequence in which the local heart flow is oscillatory, with no net transport anteriorly or posteriorly. Additionally, the primary bubble movement in the dorsal heart follows a diagonal trajectory (left side), evidencing a bend in the heart tube. Click here for file

Additional file 6 **Possible entrance of a bubble through an incurrent ostium in the heart of the grasshopper *Schistocerca americana *I.** Movie depicts the grasshopper's 5th abdominal segment in lateral view, with dorsal oriented upward and anterior to the right. Field of view is 1.3 × 0.9 mm. The dorsal edge of the abdomen can be seen near the top. The diagonal lines in the upper left corner are margins of the x-ray transparent Kapton tube. In this sequence, the bubble of interest appears to begin its trajectory outside the heart in the pericardial sinus; it moves dorsally and then anteriorly to the ostium opening; it then enters the heart and is swept anteriorly along with the flow. Although we interpret this trajectory as a heart entrance event, other possibilities remain. Click here for file

Additional file 7 **Possible entrance of a bubble through an incurrent ostium in the heart of the grasshopper *Schistocerca americana *II.** Movie depicts the grasshopper's 6th abdominal segment in lateral view, with dorsal oriented upward and anterior to the right. Field of view is 1.3 × 0.9 mm. The diagonal line in the lower left corner is a margin of the x-ray transparent Kapton tube. This sequence demonstrates a second potential ostial entrance event. Here, the bubble of interest appears to begin its trajectory outside the heart in the pericardial sinus; it moves posteriorly, dorsally, and then anteriorly to the ostium opening; it then enters the heart in a large movement to the upper right. As in Additional file 6, although we interpret this trajectory as a heart entrance event, other possibilities remain. Click here for file
